# Spatially Varying Coefficient Inequalities: Evaluating How the Impact of Patient Characteristics on Breast Cancer Survival Varies by Location

**DOI:** 10.1371/journal.pone.0155086

**Published:** 2016-05-05

**Authors:** Jeff Ching-Fu Hsieh, Susanna M. Cramb, James M. McGree, Nathan A. M. Dunn, Peter D. Baade, Kerrie L. Mengersen

**Affiliations:** 1 Mathematical Sciences, Queensland University of Technology, Brisbane, Queensland, Australia; 2 Cancer Research Centre, Cancer Council Queensland, Brisbane, Queensland, Australia; 3 Preventive Health Unit, Department of Health, Brisbane, Queensland, Australia; INRS, CANADA

## Abstract

An increasing number of studies have identified spatial differences in breast cancer survival. However little is known about whether the structure and dynamics of this spatial inequality are consistent across a region. This study aims to evaluate the spatially varying nature of predictors of spatial inequality in relative survival for women diagnosed with breast cancer across Queensland, Australia. All Queensland women aged less than 90 years diagnosed with invasive breast cancer from 1997 to 2007 and followed up to the end of 2008 were extracted from linked Queensland Cancer Registry and BreastScreen Queensland data. Bayesian relative survival models were fitted using various model structures (a spatial regression model, a varying coefficient model and a finite mixture of regressions model) to evaluate the relative excess risk of breast cancer, with the use of Markov chain Monte Carlo computation. The spatially varying coefficient models revealed that some covariate effects may not be constant across the geographic regions of the study. The overall spatial patterns showed lower survival among women living in more remote areas, and higher survival among the urbanised south-east corner. Notwithstanding this, the spatial survival pattern for younger women contrasted with that for older women as well as single women. This complex spatial interplay may be indicative of different factors impacting on survival patterns for these women.

## Introduction

The association between risk factors and small-area health outcomes of breast cancer patients is of key interest. Previous studies have used Bayesian spatial regression models to examine small-area variation in relative survival for breast cancer in Spain [1] and Australia [2–4]. In the latter studies, a range of patient characteristics, including women’s age at diagnosis, Indigenous status, partner status, tumour stage at diagnosis, occupation, detection method and area of residence were shown to be associated with breast cancer relative survival in Queensland, Australia [2–4]. In general, patients who were older, Indigenous, diagnosed with an advanced stage tumour or living in a remote area were at higher breast cancer mortality risk. Women with lower breast cancer mortality risk included patients who had participated in a breast cancer screening program, had a tumour diagnosed early, while it was still localised, or were not in the labour force or had a partner (married or defacto). In addition, there was evidence of spatial inequalities in breast cancer patient survival across Queensland, often with poorer survival in more remote areas.

In all of these models the regression coefficients were assumed to have a constant influence across the entire study region, although we have previously [4] examined a potential spatially varying effect for one particular predictor variable, the method of detecting breast cancer. In these constant coefficient models, the spatial nature of the data is captured through the inclusion of a single random effect that takes into account similarities between neighbouring regions, thus inducing a type of flexible local smoothing [2–4]. Determining if the association of key factors varies over small-areas would help to better describe and understand the varying survival rates in breast cancer among women in Queensland. In addition, as the cancer data are collected over space, it is useful and important to analyse the spatial association between covariates and health outcomes. Thus, more sophisticated spatial models are applied to analyse the spatial cancer data.

In this paper we apply a Bayesian spatial regression model with spatially varying coefficients to examine the potentially spatial varying effects of breast cancer risk factors. Hastie and Tibshirani [5] introduced the varying coefficient terms to accommodate a particular type of interaction between explanatory variables. This interaction takes the form of *β*(*s*)*x*, where the coefficient *β* of the explanatory variable *x* is varying smoothly according to another explanatory variable *s*, which is generally a continuous variable such as time or space. Numerous authors have studied varying coefficient models and found the model to be flexible and appealing for investigating dynamic patterns in the data [6–10]. The varying coefficient model provides a clearly interpretable approach for modelling the dynamic spatial relationship between the covariate and response variable.

Another type of model, the finite mixture of regressions model, has a similar capability to incorporate the changing influence of covariates on the response across a spatial domain [11, 12]. However, in contrast to the varying coefficient model, the finite mixture of regressions model separates the data records into a number of subsets and analyses each of these subsets as a separate component. This allows the vector of regression coefficients to vary from component to component [13], where in the spatial scenario the components represent clusters of geographic regions. For comparison, we also apply a Bayesian finite mixture of regressions models to the Queensland breast cancer dataset [12]. If the data support a finite mixture of regressions model to varying from clusters of geographic location, it may then be of interest to consider if the clusters reveal any unobserved covariate effect that could influence the health outcome.

In summary, this study aims to evaluate spatial variation in the factors influencing relative survival from breast cancer across the diverse geographic and demographic regions of Queensland, Australia, using three different modelling strategies, in which the spatial variation is modelled via a single random effect, through varying coefficients or through mixture components. In other words, this study aims to identify specific subgroups of women diagnosed with breast cancer whose survival outcomes depend on where they live. It is likely the results will inform the optimal design and provision of health resources aimed at addressing inequalities in breast cancer survival according to geographical location and sociodemographic characteristics.

## Materials and Methods

### Study cohort

All Queensland women aged less than 90 years and diagnosed with an invasive breast cancer (ICD-O-3 code = C50) between 1 January 1997 to 31 December 2007 and followed up to 31 December 2008 were included in the study. The datasets from Queensland Cancer Registry (QCR) and BreastScreen Queensland (BSQ) were linked by BSQ staff using a deterministic matching process with over 90% matching completeness.

Patient records were anonymized and de-identified prior to data extraction and analysis. Ethics approval was granted by the Human Research Ethics Committee of Queensland University of Technology (approval number: 1100000036). Access to the data was provided by Queensland Health under the Public Health Act 2005 (RD003676).

Cases were excluded (<1%) for patients with missing information, including age at diagnosis, geographic location, and those who were identified at autopsy or by death certificate only, or who had a survival time of less than one day.

### Explanatory variables

The study variables (see Table 1) included age group at diagnosis, Indigenous ethnicity, partner status at diagnosis, tumour stage at diagnosis, BreastScreen program participant indicator and geographic location information. The screen- and interval-detected breast cancer patients from the BSQ screening program were collapsed into a single category and compared to those who did not participate in the BSQ program, to form a binary BSQ participant indicator.

**Table 1:** Posterior estimates of relative excess risk (RER) of mortality across Queensland, 1997–2008.

		Median RER [95% CrI^a^] (unless otherwise specified)			
		Spatial Regression	Varying Coefficient		Finite Mixture
Factors	N		Median RER	VC range^b^	Cluster P^c^ = 0.998
**Age at diagnosis (years)**					
<40	1428	0.90 [0.77, 1.06]	0.84 [0.70, 1.01]	[0.520, 1.66]	0.90 [0.77, 1.06]
40–49	4669	0.82 [0.72, 0.93]	0.83 [0.71, 0.95]	[0.601, 2.10]	0.82 [0.72, 0.93]
50–59	6443	1.00	1.00	—	1.00
60–69	5545	1.12 [0.98, 1.27]	1.11 [0.95, 1.28]	[0.636, 1.46]	1.12 [0.98, 1.27]
70–89	5681	1.46 [1.29, 1.66]	1.45 [1.25, 1.68]	[0.652, 1.40]	1.47 [1.30, 1.66]
**Indigenous Status**					
Indigenous	257	1.83 [1.40, 2.37]	1.63 [1.12, 2.32]	[0.718, 1.72]	1.83 [1.39, 2.36]
Non-Indigenous	20529	1.00	1.00	—	1.00
Unknown	2980	0.03 [0.01, 0.07]	0.02 [0.01, 0.05]	[0.731, 1.74]	0.03 [0.01, 0.07]
**Partner Status**					
Has partner	14801	1.00	1.00	—	1.00
Single	1441	1.25 [1.07, 1.46]	1.29 [1.08, 1.56]	[0.594, 1.79]	1.26 [1.07, 1.46]
Widowed/Divorced/Separated	6787	1.38 [1.25, 1.51]	1.38 [1.24, 1.54]	[0.739, 1.36]	1.38 [1.26, 1.51]
Unknown	737	0.38 [0.16, 0.70]	0.19 [0.03, 0.52]	[0.220, 21.75]	0.38 [0.17, 0.69]
**Tumour Stage**					
Localised (Stage I)	11517	1.00	1.00	—	1.00
Advanced (Stage II, III, IV)	10699	4.23 [3.70, 4.87]	4.23 [3.68, 4.91]	[0.798, 1.25]	4.23 [3.71, 4.85]
Unknown	1581	14.03 [12.26, 16.77]	14.53 [12.35, 17.20]	[0.690, 3.00]	14.29 [12.32, 16.66]
**BSQ Participant**					
Yes	9745	1.00	1.00	—	1.00
No	14052	1.91 [1.71, 2.12]	1.96 [1.74, 2.21]	[0.927, 1.06]	1.92 [1.72, 2.14]
**DIC**		34797	34795		34861
**pD**		113	345		177
**PPC**		98.61%	98.64%		99.99%

^*a*^Abbreviations: CrI = Credible interval, N = Number of patients, DIC = Deviance information criterion, pD = Effective number of parameters, PPC = posterior predictive check.

^*b*^Exponentiated median varying coefficient (VC) values (exp(*δ*_*i*_)) of 478 SLA.

^*c*^Mixing probability of SLA been allocate in the cluster.

Geographic location information of the 478 Statistical Local Areas (SLAs) was based on the 2006 version of the Australian Standard Geographical Classification covering the whole of Queensland without gap or overlap.

### Response variable

The response variable is the observed number of deaths (*d*_*ϵ*_) due to any cause among the cohort of eligible Queensland breast cancer patients for each stratum (*ϵ*). Stratum refers to the covariate categorisation. It is the different combination of predictor categories for each observation. This was modelled by a generalized linear model with a Poisson likelihood,

$$\begin{array}{c} d_{\epsilon }∼Poisson\left(\mu_{\epsilon }\right). \end{array}$$ (1)

In a relative survival model, the expected number of deaths (*μ*_*ϵ*_) can be modelled by

$$\begin{array}{c} \mu_{\epsilon }=d_{\epsilon }^{*}+y_{\epsilon }\times \exp\left(\eta_{\epsilon }\right), \end{array}$$ (2)

where $d_{\epsilon }^{*}$ is the expected numbers of non-breast cancer deaths for each stratum of the eligible cohort [2], *y*_*ϵ*_ is the person-time at risk and *η*_*ϵ*_ is the excess hazard. The expected number of non-breast cancer deaths were calculated using population mortality rates for each SLA that smoothed over neighbouring SLA data to provide greater stability.

The exponential of the individual components of *η*_*ϵ*_ provides the relative excess risk of death (RER) for the corresponding model variables. The specific model equations for the excess hazard are described below.

### Statistical models

As an exploratory analysis, each single predictive variable was separately fitted in the spatial regression, finite mixture of regressions and varying coefficient models. This was a simple process to assess the possibility of confounders or missing interactions in the data. Three models were then fitted using the full set of covariates.

#### Spatial regression model

The excess hazard (*η*_*ϵ*_) is modelled by

$$\begin{array}{c} \eta_{\epsilon }=\alpha_{t}+\beta x+u_{i}+v_{i} \end{array}$$ (3)

where *α*_*t*_ is the *t*^*th*^ intercept that varies by follow-up interval for *t* = 1, 2, …, 12; *β* is the coefficient vector associated with the vector of predictor variables **x** listed in the ‘Explanatory variables’ section; and *u*_*i*_ and *v*_*i*_ represent the spatially structured and unstructured random effects respectively for the *i*^*th*^ SLA, *i* = 1, 2, …, 478.

The model components *α*_*t*_, *β* and *v*_*i*_ were all assigned a zero mean Gaussian distribution with a flat hyperprior distribution Gamma(0.005, 0.5), parameterized in terms of the shape and inverse scale parameters, for the precision. The random effect term *u*_*i*_ was assigned an intrinsic conditional autoregressive (CAR) [14] prior distribution with a Gamma(0.5,0.005) hyperprior distribution for the precision [15, 16].

The exponential of each of the components in Eq (3) gives the estimated RER of the corresponding variables (i.e. exp(*α*), exp(*β*) and exp(*u*_*i*_ + *v*_*i*_)).

#### Varying coefficient model

In this model the excess hazard (*η*_*ϵ*_) in Eq (3) is modified as follows:

$$\begin{array}{c} \eta_{\epsilon }=\alpha_{t}+\left(\beta +\delta_{i}\right)x+u_{i}+v_{i}. \end{array}$$ (4)

The modification allows for an additional spatial random effect term *δ*_*i*_ for each of the covariates. The prior distributions for all other variables in Eq (4) are the same as for the spatial regression model variables in Eq (3). The additional spatial random effect term *δ*_*i*_ for each SLA was assigned a multivariate intrinsic Gaussian CAR prior distribution with a precision matrix described by a Wishart distribution *Σ* ∼ Wishart(*Q*, *k*), where *Q* is a *k* × *k* identity matrix, to allow for correlation among the *k* variables.

Models with no additional spatially structured random effect (*u*_*i*_) in Eq (4) were also considered.

#### Finite mixture of regressions model

In this model the excess hazard (*η*_*ϵ*_) in Eq (3) is modified as follows:

$$\begin{array}{c} \eta_{\epsilon }=\alpha_{tj}+\beta_{j}x+u_{ij}+v_{ij}\;\text{with}\;\text{probability}\;\pi_{j} \end{array}$$ (5)

$$\begin{array}{c} \pi_{j}∼Dirichlet\left(\phi_{1},...,\phi_{J}\right) \end{array}$$ (6)

Here the excess hazard (*η*_*ϵ*_) has been allocated into a number of clustered SLA subgroups *j* = 1, …, *J*. The total number of subgroups was allowed to range from *J* = 2 to 6. The upper bound of 6 was chosen based on previous studies that SLAs can be collapsed into 4–5 spatial regions based on their geographic characteristics such as socio-economic status and area remoteness index [3, 17, 18]. Each mixture subgroup of excess hazard was assigned a mixing probability of *π*_*j*_ where 0 < *π*_*j*_ < 1 and $\sum_{j=1}^{J}\pi_{j}=1$. The 478 SLAs were assigned into different geographic subgroups (*j*) by a multinomial distribution with parameters (*π*_1_, …, *π*_*J*_). These parameters were assigned a flat Dirichlet prior as in Eq (6), with all concentration parameters (*ϕ*_1_, …, *ϕ*_*J*_) set equal to 1. The model components *α*_*tj*_, *β*_*j*_ and *v*_*ij*_ were all assigned a zero mean Gaussian distribution with a hyperprior distribution Gamma(0.005, 0.5), parameterized in terms of the shape and inverse scale parameters, for the precision.

Models with no spatially structured (*u*_*ij*_) or unstructured (*v*_*ij*_) random effect in Eq (5) were also examined.

Alternative prior representations for *π*_*j*_ Eq (6) were also evaluated, including the Dirichlet distribution with varying concentration parameter (*ϕ*) and the *π*_*j*_ ∼ *GEM*(*ϕ*) distribution, after Griffiths, Engen and McCloskey, as in Pitman (2002) [19]. As these methods did not alter the results, the simplest method, namely a Dirichlet distribution with a vague constant concentration parameter (*ϕ*_1_, …, *ϕ*_*J*_ = 1), was implemented.

### Model computation

All models were estimated using Markov chain Monte Carlo (MCMC) via WinBUGS v1.4.3 [20] interfaced with R v2.14.1 [21]. All models were run with 2 chains and a thinning factor of two. The spatial regression and varying coefficient model was run for 30,000 iterations with 20,000 for burn-in, so 5,000 iterations were retained for inference. The mixture of regressions model was run for 300,000 iterations with a burn-in of 290,000 and thinning factor of two, which left 5,000 iterations for inference. The larger burn-in was required to allow the mixture components to become well differentiated. Model convergence was examined by means of the Gelman-Rubin convergence diagnostic [22], trace plots, posterior density plots and autocorrelation plots.

### Model evaluation

As all the fitted models used the same dataset, the Deviance Information Criterion (DIC) was used to determine how well each model fits the analysed data, with smaller values indicating better model fit [23]. The DIC is the sum of the mean deviance and an estimate of the effective number of parameters (pD), with the complexity of a model increasing with ascending pD values and vice versa. Posterior predictive checks (PPC) [23] were applied to examine the adequacy of the model predictions compared with the observed data. The exponentiated regression coefficients were considered to be substantively raised or lowered (for continuous variables) or different from their respective baseline covariate category (for categorical variables) if the 95% credible interval (CrI) did not include unity [24–26].

In order to assess the spatial RER inequalities from the varying coefficient model, the random effect terms (*u*_*i*_, *v*_*i*_ and *δ*_*i*_) were mapped to visually identify any spatial patterns. A set of common fixed cut-off values (<0.77, 0.91, 1.10, 1.30, 1.30+) was also used to divide the mapped values into five separate groups to reduce the likelihood of reporting spurious differences and to facilitate comparison of spatial patterns between maps [18, 27]. The SLA-specific RER (exp(*u*_*i*_ + *v*_*i*_)) map gives the pattern of overall spatial RER inequalities of the baseline group of patients (i.e. aged 50–59, non-Indigenous, had a partner, localised (stage I) tumour and BSQ participant) compared to the Queensland average (value of 1). The exponentiated spatially varying coefficient (SVC) effects (exp(*δ*_*i*_)) for each predictive variable were also mapped to reveal spatial varying patterns (S1 and S2 Figs). In order to compare across various SVC effects, the combined spatial variation (exp(*δ*_*i*_ + *u*_*i*_ + *v*_*i*_)) with regard to the covariate-specific Queensland average (value of 1), called the relative spatially varying coefficient (RSVC), was also calculated and mapped. Maps of the posterior probability that the RSVC exceeded unity were created to assess the RSVC pattern with the incorporation of posterior uncertainty. Applying the cut-off suggested by Richardson et al. [28], a probability higher than 0.8 or lower than 0.2 indicates there is little uncertainty that this differs from unity.

All model convergence measures indicated acceptable convergence, as the Gelman-Rubin test statistics were close to unity, trace plots showed good mixing of chains and autocorrelation plots diminished rapidly.

## Results

Table 1 presents all the spatially constant median RER estimates for the spatial regression model, the range of estimates for varying coefficient model, and the analogous estimates for the two component finite mixture of regressions model with mixing probabilities for each cluster. The other finite mixture of regressions model with 3–6 components were having similar results as to the two component model, and hence the results are not shown. The estimated constant covariate results for the spatial regression model are the same as in the previous study [3]. Higher relative excess risk of mortality was observed in older patients, of Indigenous ethnicity, were single, widowed, divorced or separated, with an advanced tumour or not participating in a screening program. Very similar constant covariate effects were also observed in the varying coefficient and finite mixture of regressions model.

For the finite mixture of regressions model, only a single cluster had substantial weight, with a mixing probability of almost 1 (Cluster P = 0.998). Moreover, the median RER and 95% credible interval of this cluster were almost identical to the values obtained for the spatial regression model. This suggests that there is no evidence of geographic clusters in which the relative survival from breast cancer differs. The removal of both spatially structured and unstructured random effects from the mixture model did not provide a substantial improvement to the model fit (Table 2).

**Table 2:** Model reduction comparison.

	Varying coefficient	Finite Mixture
**Full model**		
DIC ^a^	34795	34861
pD	345	177
**No spatial structured effect**		
DIC	34814	—
pD	335	—
**No spatial structured & unstructured effect**		
DIC	—	34823
pD	—	36

^*a*^Abbreviations: DIC = Deviance information criterion, pD = Effective number of parameters.

**Fig 1:**
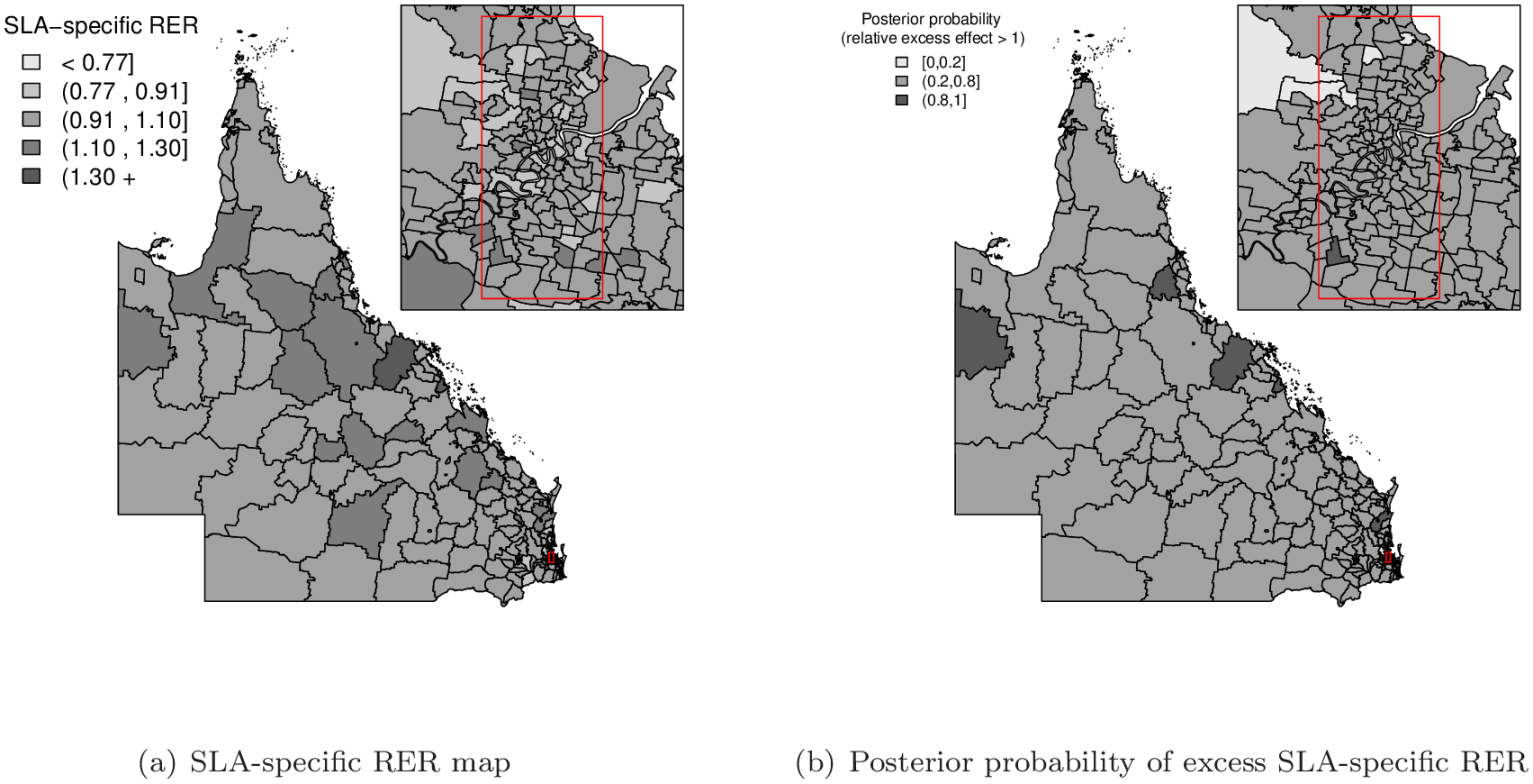
SLA-specific relative excess risk (RER) (exp(*u*_*i*_ + *v*_*i*_)) map for the varying coefficient model.

**Fig 2:**
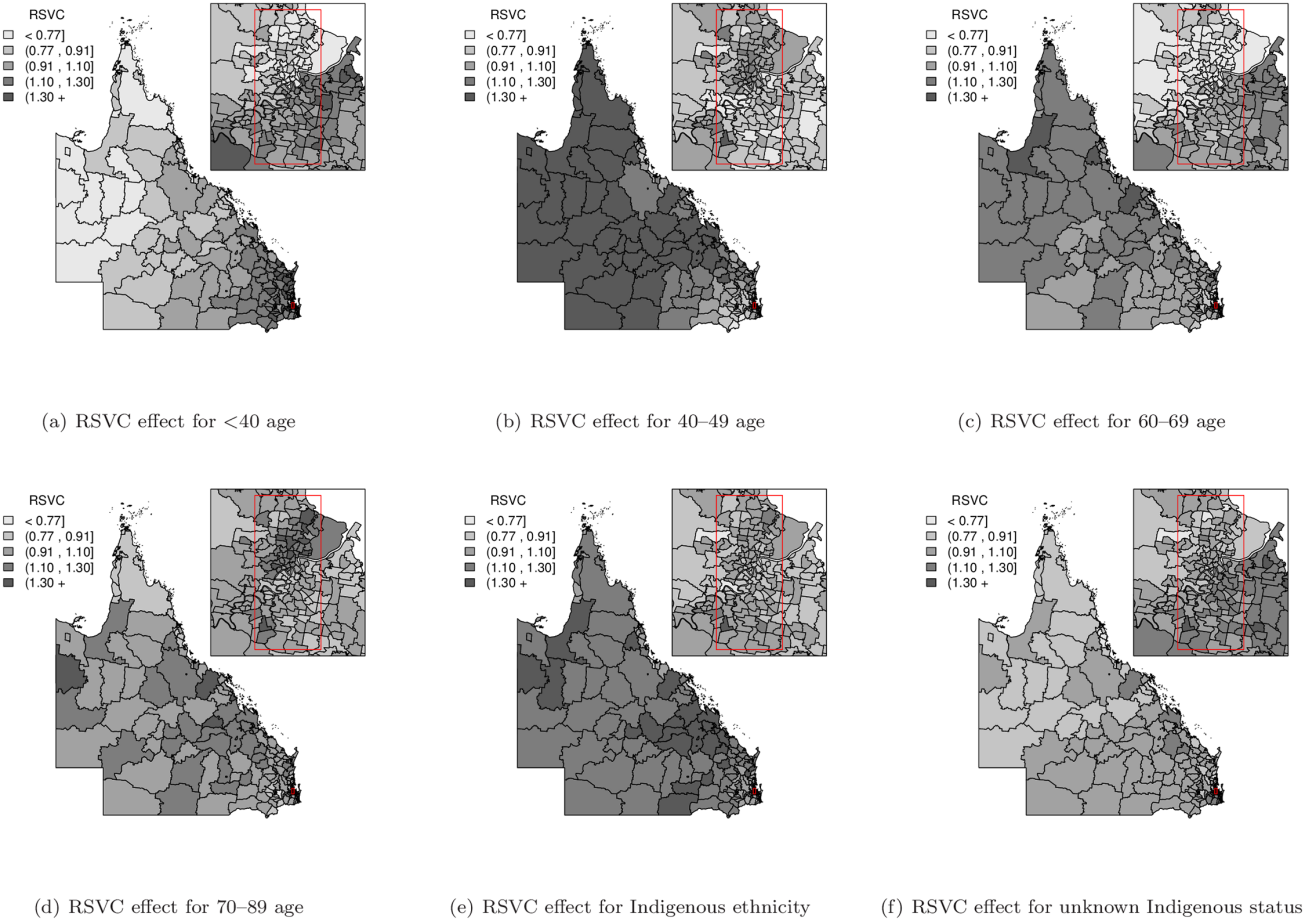
Relative spatially varying coefficient (RSVC = exp(*δ*_*i*_ + *u*_*i*_ + *v*_*i*_)) effect maps for the age at diagnosis and Indigenous status variables.

**Fig 3:**
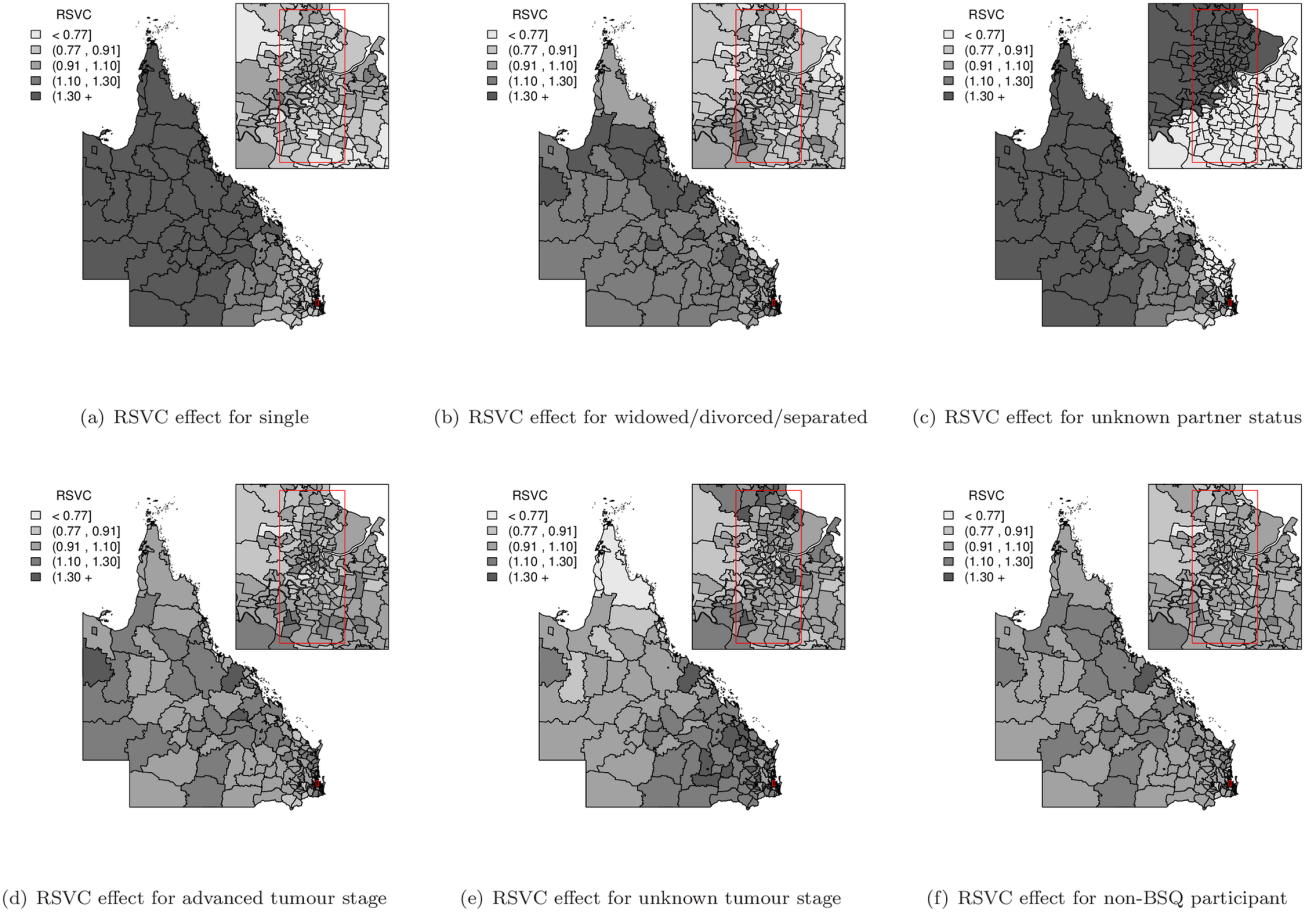
Relative spatially varying coefficient (RSVC = exp(*δ*_*i*_ + *u*_*i*_ + *v*_*i*_)) effect maps for the partner status, tumour stage and BSQ participant variables.

In contrast, the varying coefficient model provided a similar fit to the data as the spatial regression model, as evidenced by the DIC statistics, but indicated distinct differences in spatial variation across the region, shown by the range of varying coefficient (VC) for the model parameters (Table 1). The general pattern of spatial variation in Fig 1 shows an increased trend of SLA-specific RER towards the north and west of Queensland, which is most similar to the SLA-specific RER maps for the spatial regression model and finite mixture of regressions model (S3 Fig), but with reduced spatial variation. The maps of estimated RSVC effects shown in Figs 2 and 3 illustrate different spatial patterns among the predictive variable categories compared to the SLA-specific RER map of combined spatially structured (*u*_*i*_) and unstructured (*v*_*i*_) random effects in Fig 1. While some of the relative spatially varying coefficients in Figs 2 and 3 show a similar increasing trend across Queensland, there are other predictive variable categories that show different RSVC trends. Of particular interest is the RSVC map for those <40 years of age at diagnosis (Fig 2(a)), which has an opposite RSVC trend that increases toward the south-east of Queensland with supporting evidence of corresponding excess posterior probability map of RSVC in S4 Fig. Moreover, the predictive variable category of women in the age group of 40–49 years (Fig 2(b)) and those that were single at diagnosis (Fig 3(a)) had an obvious increasing trend from south-east to north Queensland, which were clearly supported by the corresponding posterior probability maps in S4 and S5 Figs. The exclusion of spatially structured random effects (*u*_*i*_) of the response variable from the varying coefficient model did not improve the outcome and thus the full varying coefficient model was preferred (Table 2).

Sensitivity analyses were conducted by changing the parameters *α*_*t*_, *β*, *u*_*i*_ and *v*_*i*_ to Gamma(0.5,0.5). The sensitivity to the choice of prior for *δ*_*i*_ was assessed by imposing different diagonal values (0.01,0.5,1 and 2) on the *Q* matrix of the hyperprior Wishart distribution. These analyses showed that the RSVC trends were not sensitive to the choice of distribution on *α*_*t*_, *β* and *v*_*i*_ but, as anticipated, substantive changes to the precision of the spatially structured random effect *u*_*i*_ may results in increased RSVC values.

## Discussion

Using a population-based cohort of Queensland women diagnosed with breast cancer, this study has presented the use of spatial regression, spatially varying coefficient regression models and finite mixture of regressions models to quantify how the impact of patient characteristics and clinical factors on relative survival outcomes varies by geographical location. This study found that the varying components model provided similar fit for our data compared to the spatial regression model, but with additional spatial varying coefficient information, and both models fit the data better than the finite mixture of regressions model. Allowing the model parameters to change by geographical location provides a much greater understanding of the impact of important variables on survival. These reduced the overall spatial variation in the SLA-specific RER map of combined spatially structured (*u*_*i*_) and unstructured (*v*_*i*_) random effects in Fig 1, compared to the spatial regression model and finite mixture of regressions model maps (S3 Fig). The different spatial patterns can be mapped, as illustrated in Figs 2 and 3; see also S1 and S2 Figs. To the best of our knowledge, this is the first study to use varying coefficient models to explore the spatial impact that multiple patient and clinical factors have on breast cancer relative survival, especially across the diverse geographic and demographic regions of Queensland, Australia.

The spatial regression model quantified overall spatial inequalities through spatially structured and unstructured random effects by assuming that they were constant across all cohort subgroups. This model found that, on average, younger women, those with non-Indigenous ethnicity, had a partner, were diagnosed with localised stage tumour or participated in the BSQ program had better survival outcomes. However, a similar fit to the data was provided by the varying coefficient model, which removed this assumption of constant spatial coefficients. These models, and the corresponding maps of the relative spatially varying coefficient and posterior probabilities, suggest that each patient subgroup (predictor category) has their own specific spatial pattern. As such, there is a different pattern of spatial variation between different age groups, with better survival for women less than 40 years of age at diagnosis in remote areas compared with urban areas, whereas for women diagnosed over 40 years of age the spatial survival differential is reversed. In other words, younger women generally have better survival when they live in remote areas, rather than in urban areas. However, there are exceptions to this, with some SLAs in remote areas having average RSVCs while some SLAs in Brisbane have low RSVCs.

It is unclear what unobserved spatial variable would account for a changing effect of age on survival by spatial location. However, previous studies, including in Queensland, have reported that younger women were more likely to be diagnosed with advanced breast cancer, with their tumours tending to be larger, metastasised and less well differentiated compared with older women [17, 29, 30]. These characteristics are consistent with breast cancers among younger women being diagnosed as a result of symptoms rather than through participation in mammography screening. However our results are adjusted for a broad measure of spread of disease at diagnosis, so apart from the possibility of residual confounding, it is unlikely that differences in diagnostic patterns can explain this result. A recent paper [2] quantified that around 7% of breast cancer deaths within 5 years of diagnosis could be attributed to non-diagnostic factors such as treatment, rehabilitation, environmental factors such as area disadvantage, and other patient characteristics including comorbidities, so it is possible that at least some of these also vary by age and location. While only a relatively small proportion of breast cancers are diagnosed in women under 40 years of age, the impact of a diagnosis of breast cancer on these younger women in terms of loss of life expectancy is much greater than for older women [31]. Clearly, further investigation is required to better understand the possible mechanisms which are driving this age differential in spatial patterns.

The sociodemographic group of single women at diagnosis has a clear spatial trend that can be observed in Fig 3(a) with its corresponding posterior probability map in S5 Fig. The spatial pattern suggests that single women living in remote areas are “at a disadvantage”, compared with corresponding women living in urban areas. In Queensland, often residents of remote areas have lower breast cancer survival than their urban counterparts [2, 3], despite similar mammography screening rates [32]. Marriage, or having a partner, has been associated with improved breast cancer survival outcomes, but so has social support [33]. Reason for the observed remoteness effect are unclear, but could include differing levels of support from social networks, potentially impacted by distance in sparsely populated areas. Alternatively, it is possible that barriers to accessing treatment for remote women have greater impact without the support of a partner in managing responsibilities. In some studies, unmarried cancer patients have had a significantly higher risk of presentation with metastatic cancer and lower likelihood of receiving optimal treatment [34].

Confusing the picture, however, was the lack of evidence for multiple clusters, or aggregations of SLAs, when applying the finite mixture of regressions model. Within our study cohort, the posterior distributions of the covariate effects and the predicted RER were statistically indistinguishable in all of the modelled mixture components, and the estimated mixing probabilities in the mixture model placed almost all the weight on a single component. Therefore, the spatially-varying coefficients identified through the varying coefficient model could not be represented as geographically contiguous spatial clusters of SLA. Possible explanations could include a different contiguous pattern in the covariate space at the SLA level, or unmeasured spatial effects at the individual level that are reflected in a more complex manner at the SLA level. This is possible given the relatively crude measure of socio-economic status at an aggregate level, for example. An alternative explanation is that the use of identical model structures for each of the mixture components impeded our ability to capture any real unmeasured spatial covariate. The better fit provided by the spatially varying model compared to the other alternatives in this study, combined with the posterior probability maps, add to the evidence of there being genuine differences in spatial patterns in survival across age for women diagnosed with breast cancer, but no evidence of geographically contiguous clustering in survival outcomes.

Several advantages may result from using the complex spatially varying model. This model has the advantage over the more commonly used spatial model by providing more insight in to the spatial variation of all the relevant variables of interest, which would be able to show how the factor effects vary by spatial location (i.e. spatially varying coefficient), instead of assuming they are constant. In addition, it can also help to identify the change of spatial variation pattern between categories of covariate (i.e. between younger and older age groups). As such, these findings have the potential to provide greater clarity for allocating health care resources, in that they recognise that spatial inequalities may have different characteristics for different subgroups of the population.

One of the limitations of this study is the model complexity which has the drawback of the increasing needs of computational resources, especially for the finite mixture of regressions model. Another limitation is the lack of information about the tumour characteristics that would have reduced the potential for residual confounding when adjusting for spread of disease at diagnosis.

The methods applied in this paper, and insights that can be derived from the corresponding analyses, can be applied across a wide range of studies in the spatial epidemiology and other spatial fields. Overall, we found that the impact of patient demographics on breast cancer survival did vary by age and partner status, but there was no evidence that the spatial inequalities could be represented as geographically contiguous spatial clusters of SLAs. It remains a priority to better understand the reasons for these differences in spatial patterns to enable appropriate interventions and strategies to be developed to help ensure equitable and improved outcomes for all women diagnosed with breast cancer, regardless of where they live.

## Supporting Information

S1 Fig Spatially varying coefficient (SVC = exp(*δ*_*i*_)) effect maps for the age at diagnosis and Indigenous status variables. (TIF)

S2 Fig Spatially varying coefficient (SVC = exp(*δ*_*i*_)) effect maps for the partner status, tumour stage and BSQ participant variables. (TIF)

S3 Fig SLA-specific relative excess risk (RER) (exp(*u*_*i*_ + *v*_*i*_)) maps. (TIF)

S4 Fig Maps of posterior probability of excess relative spatially varying coefficient (RSVC) effect for the age at diagnosis and Indigenous status variables. (TIF)

S5 Fig Maps of posterior probability of excess relative spatially varying coefficient (RSVC) effect for the partner status, tumour stage and BSQ participant variables. (TIF)
