# Supplementary figures and images for: Spatially Varying Coefficient Inequalities: Evaluating How the Impact of Patient Characteristics on Breast Cancer Survival Varies by Location
Source: PLoS One. 2016 May 5;11(5):e0155086. doi: 10.1371/journal.pone.0155086 (PMC4857928; doi:10.1371/journal.pone.0155086)

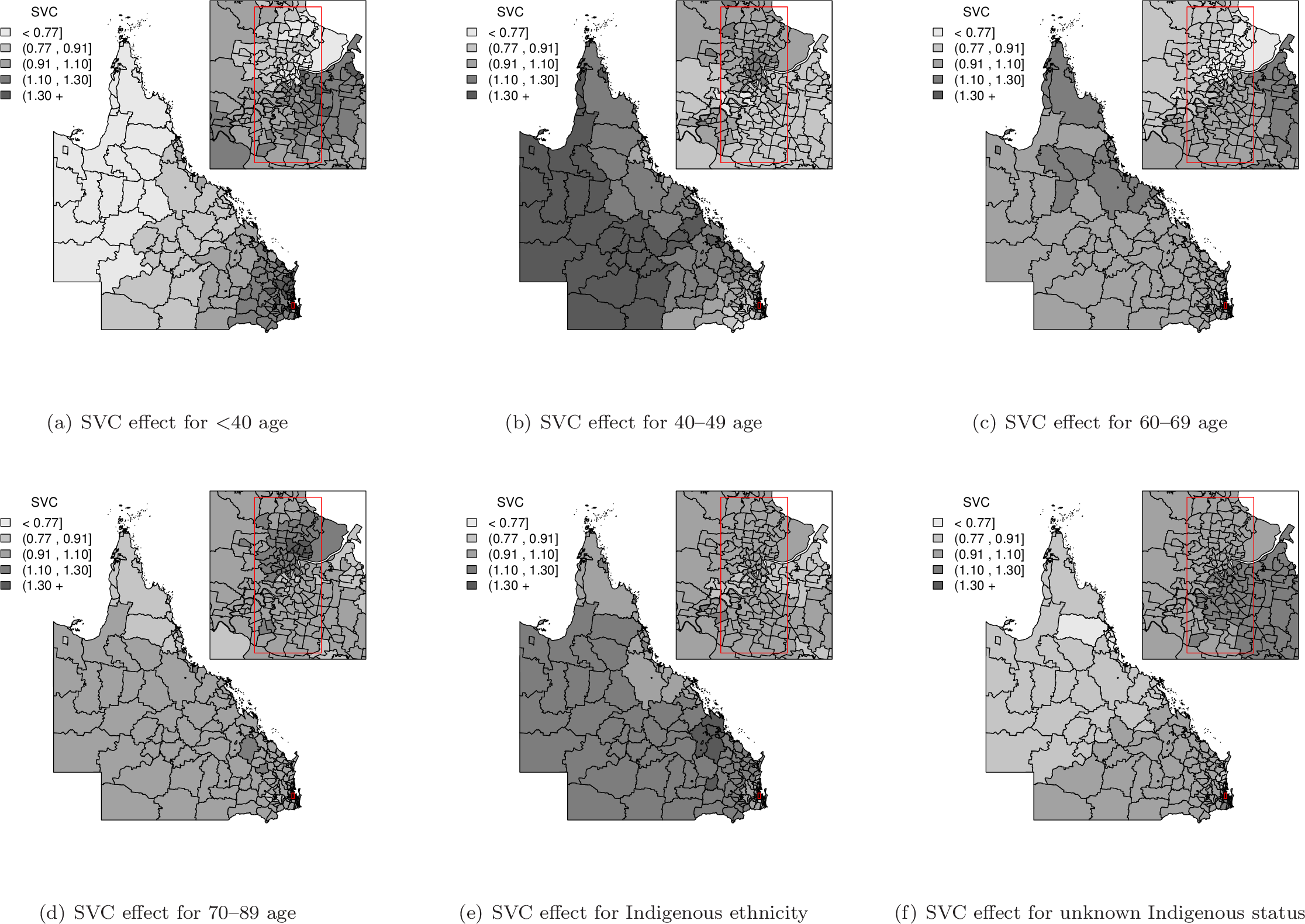

Supplement: S1 Fig — (TIF) [file pone.0155086.s001.tif]

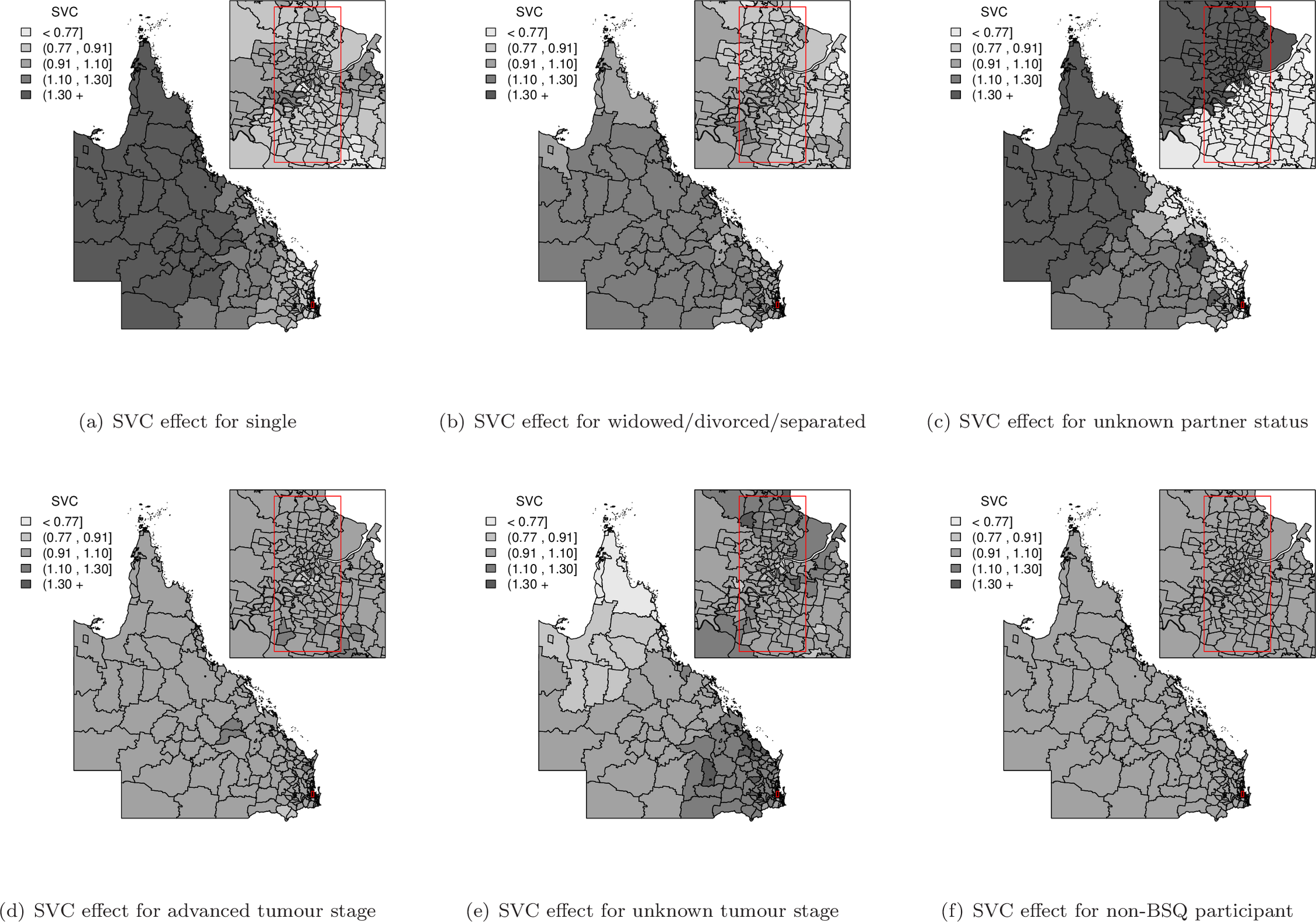

Supplement: S2 Fig — (TIF) [file pone.0155086.s002.tif]

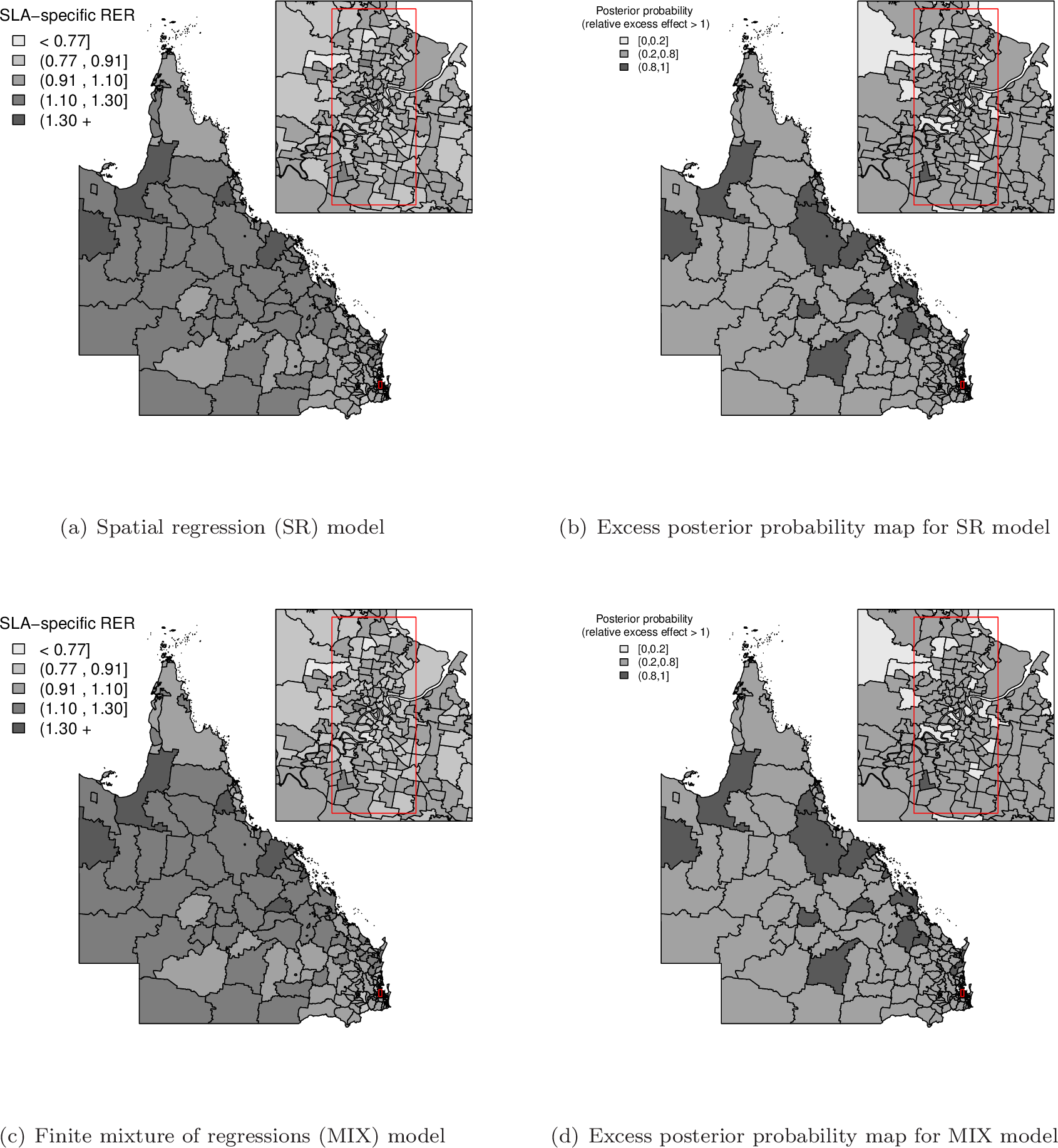

Supplement: S3 Fig — (TIF) [file pone.0155086.s003.tif]

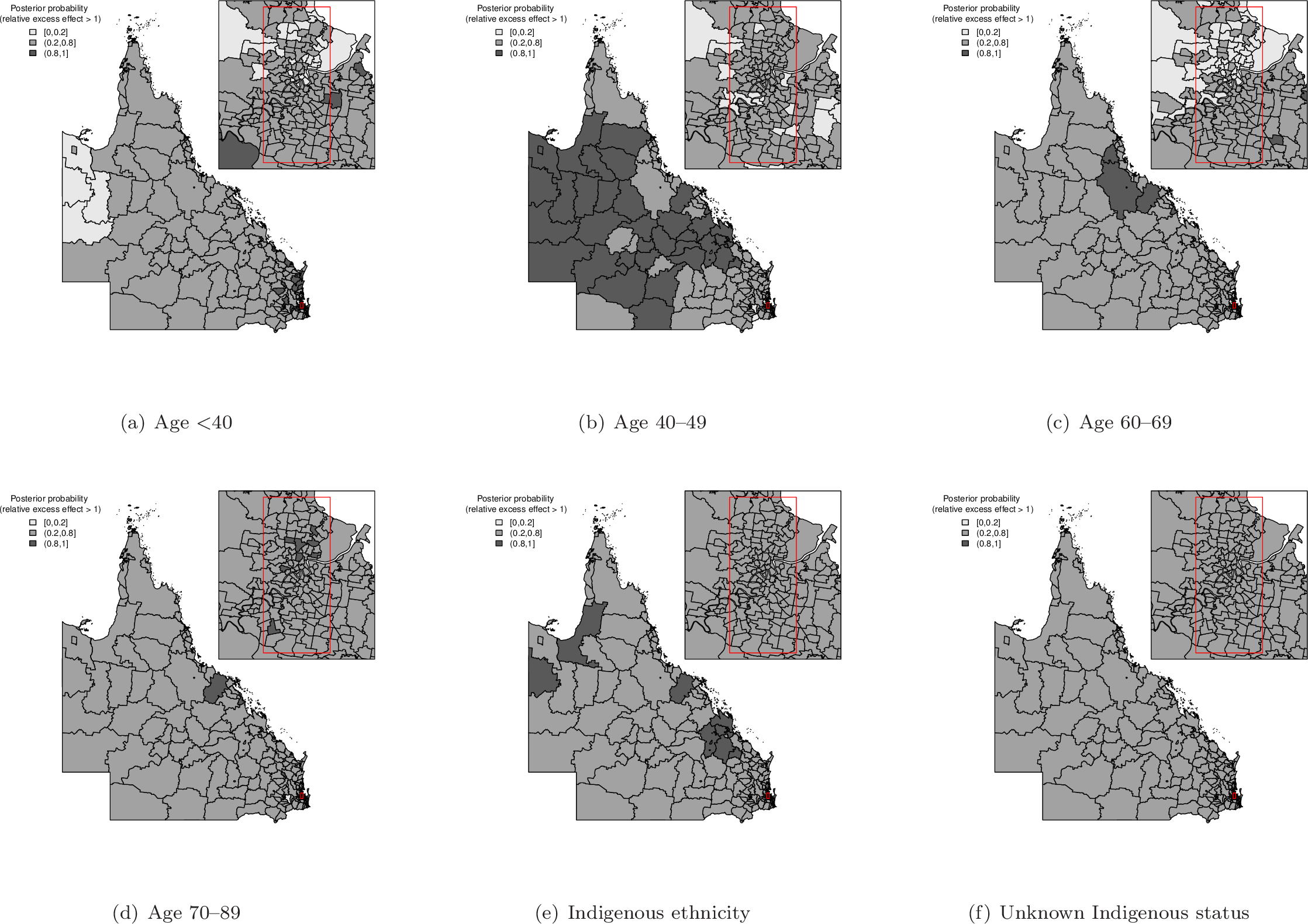

Supplement: S4 Fig — (TIF) [file pone.0155086.s004.tif]

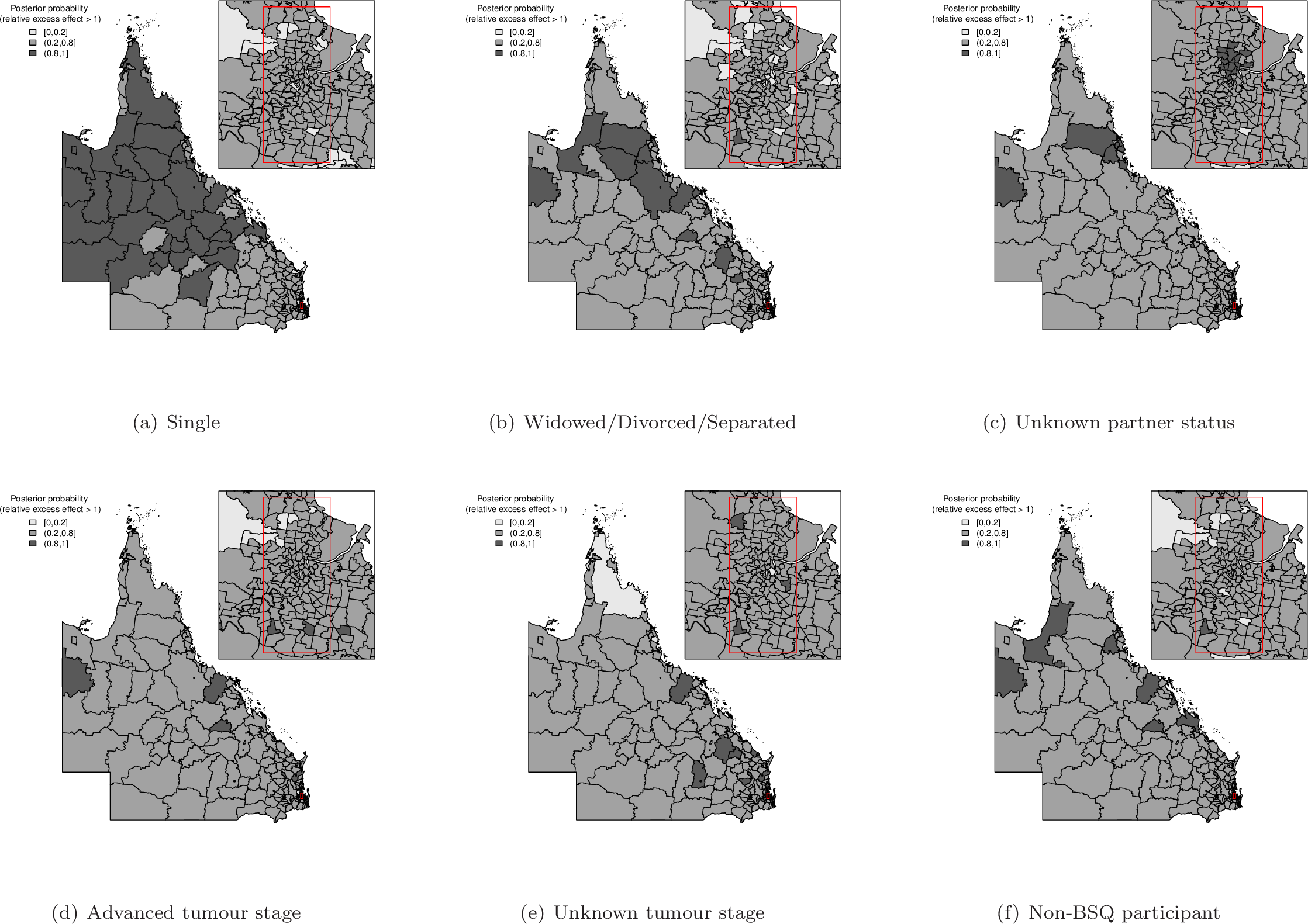

Supplement: S5 Fig — (TIF) [file pone.0155086.s005.tif]
